# A CRISPR/Cas9 screen in embryonic stem cells reveals that *Mdm2* regulates totipotency exit

**DOI:** 10.1038/s42003-024-06507-9

**Published:** 2024-07-03

**Authors:** Chen Gao, Xin Gao, Fei Gao, Xuguang Du, Sen Wu

**Affiliations:** 1 State Key Laboratory of Animal Biotech Breeding, College of Biological Sciences, China Agricultural University, Beijing, 100193 China; 2 State Key Laboratory of Animal Biotech Breeding, Institute of Animal Science, Chinese Academy of Agricultural Sciences, Beijing, 100193 China; 3 Sanya Institute of China Agricultural University, Sanya, 572025 China

**Keywords:** Totipotent stem cells, Embryonic stem cells

## Abstract

During early embryonic development, the transition from totipotency to pluripotency is a fundamental and critical process for proper development. However, the regulatory mechanisms governing this transition remain elusive. Here, we conducted a comprehensive genome-wide CRISPR/Cas9 screen to investigate the 2-cell-like cells (2CLCs) phenotype in mouse embryonic stem cells (mESCs). This effort led to the identification of ten regulators that play a pivotal role in determining cell fate during this transition. Notably, our study revealed *Mdm2* as a significant negative regulator of 2CLCs, as perturbation of *Mdm2* resulted in a higher proportion of 2CLCs. *Mdm2* appears to influence cell fate through its impact on cell cycle progression and H3K27me3 epigenetic modifications. In summary, the results of our CRISPR/Cas9 screen have uncovered several genes with distinct functions in regulating totipotency and pluripotency at various levels, offering a valuable resource for potential targets in future molecular studies.

## Abstract (web summary)

A CRISPR/Cas9 screen identities regulators about the transition of pluripotency to totipotency and revealed Mdm2 as a significant negative regulator of totipotency.

## Introduction

After fertilization, the zygote is formed through the successful fusion of an egg cell with a sperm cell. The zygote undergoes a crucial transition, initiating a novel transcriptional program to accommodate the intricate demands of subsequent embryo development. In mouse, the significant event, known as zygotic genome activation (ZGA), prominently unfolds in two-cell (2C) embryos and activates 2C-specific genes such as *Dux* and *Zscan4*^1–4^. After ZGA, the totipotent embryos later differentiate into either extra-embryonic lineages or embryonic tissues, which marks a critical point in the divergence of cell fates^5^. Accurate cellular fate determination depends on the precise coordination of gene activation for subsequent developmental phases and simultaneous suppression of genes linked to earlier stages. Indeed, the specific regulators associated with these processes have remained elusive. Consequently, deciphering the intricate regulatory network governing totipotency and pluripotency in early mammalian development represents a pivotal and compelling research endeavor.

In the culture of mESCs, a small subset referred to as 2CLCs has garnered significant attention owing to their exceptional capability to display totipotency and transcriptional features reminiscent of 2C embryos^6^. These 2CLCs were identified using the MERVL reporter and are characterized by the specific expression of transcripts of *Dux* and *Zscan4* clusters. The emergence of 2CLCs presents an excellent model system for delving into the molecular mechanisms that regulate totipotency. Recent research has revealed that certain epigenetic factors and 2C-specific genes can influence the transition to a 2C-like state in mESCs based on distinctive features shared with 2CLCs or 2C embryos, such as increased chromatin mobility, chromocenter decondensation and transcriptional characteristics^2,3,7,8^. Nevertheless, the comprehensive and precise understanding of the mechanism, particularly the processes facilitating the transition from totipotency to pluripotency, remains incomplete.

Recently, Rodriguez-Terrones et al. conducted an siRNA screen targeting epigenetic regulators, revealing the involvement of PRC1.6 and the EP400–TIP60 complex in suppressing the 2C-like state^9^. However, the inherent limitations of siRNA screens should be noted, as they often fail to achieve complete gene expression downregulation. Additionally, the screen was constrained by a limited repertoire of well-established pathways to target. This highlights the potential existence of additional, yet undiscovered factors that play a role in regulating totipotency. Later, Fu et al. employed a CRISPR/Cas9 screen to investigate the regulators of 2CLCs’ transition in mESCs with overexpressed *Dux*^10^. Notably, *Dux* expression can activate 2C genes and maintain totipotency, leading to a significant proportion of 2CLCs in mESCs, with rates reaching up to 50%. Consequently, the persistent expression of *Dux* presents a challenge in screening for factors that drive the transition from totipotency to pluripotency. This continuous *Dux* expression sustains the activation of totipotency genes, making it difficult to facilitate their exit from this state.

In order to deepen our understanding of cell fate transitions, we conducted a comprehensive genome-wide CRISPR/Cas9 screen in mESCs using the MERVL-tdTomato reporter system. Our screening identified genes linked to cell metabolism, RNA biosynthesis, and the G1/S cell cycle phase through deep sequencing of enriched sgRNA populations. Validation studies confirmed that these genes, which had not been previously reported, serve as potential regulators of the transition between totipotency and pluripotency. In particular, our research identified *Mdm2* as a negative regulator of totipotency. Disruptions of *Mdm2* led to enhanced transcriptional activity of both 2C genes, while *Mdm2* overexpression had inhibitory effects on these genes. Specifically, *Mdm2* had the ability to shorten the duration of the G1 phase, indicating the transition to a pluripotent state, and it could also elevate H3K27me3 modifications at *Dux*, which served as an indicator of exiting the totipotent state. Taken together, our research highlights the validity of our screening approach in identifying crucial regulators that govern the transition between totipotency and pluripotency.

## Results

### Genome-wide CRISPR screen to identify regulators for resistance to totipotency

**Fig. 1:**
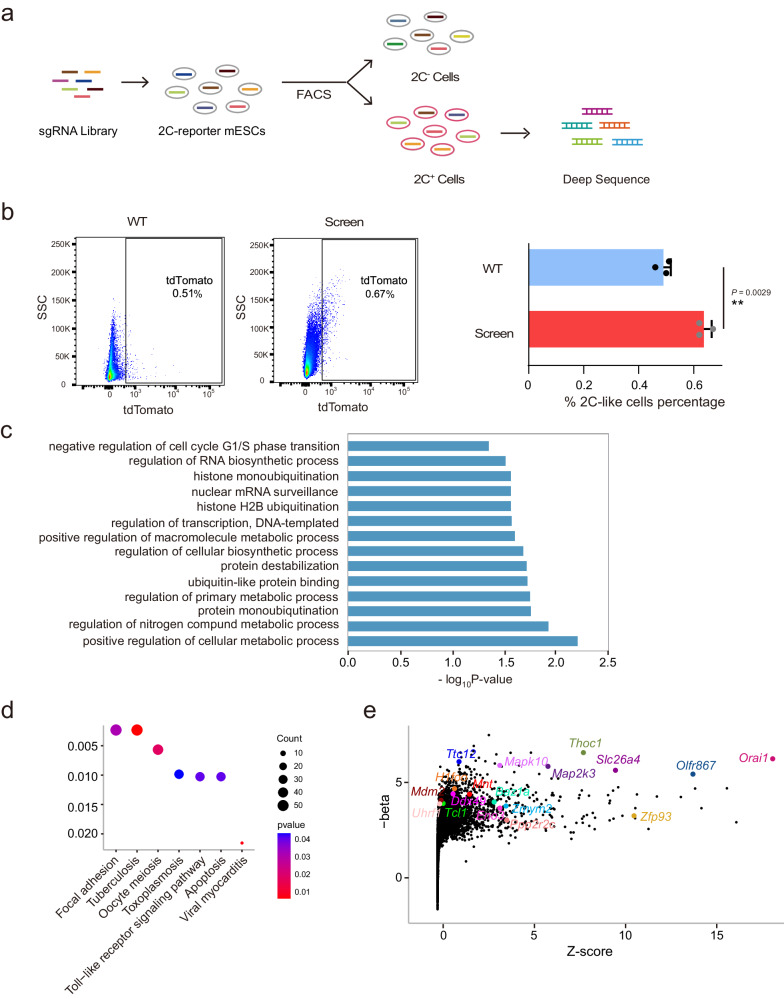
Conducting a genome-scale CRISPR knockout screening to identify novel regulators of totipotency. **a** Schematic diagram of the genome-scale CRISPR/Cas9 screen. **b** FACS analysis showing the percentage of 2CLCs after screening. The histogram presents the mean percentage of 2CLCs in WT control and screening mESCs, with n = 3 biological replicates, and the data were mean with SD. Data are analyzed by Student’s t-test. **P < 0.01. **c** Gene ontology analysis of significantly enriched genes (the Fisher test). **d** KEGG analysis of significantly enriched genes (the hypergeometric test). **e** Screening results displaying MAGeCK beta scores and Z-scores for each gene. Genes marked in the list will undergo verification.

To identify regulators that impede totipotency in mESCs, we conducted a comprehensive genome-wide CRISPR/Cas9 screen. For this screening, we developed a dual fluorescent reporter cell line, OCT4-GFP and MERVL-tdTomato, in mESCs (Supplementary Fig. 1a). These cells were denoted as OG2CT mESCs (Oct4-GFP-2C promoter-tdTomato mESCs), and they exhibited a 0.5% population of 2CLCs (Supplementary Fig. 1b, c), a proportion consistent with previous reports (Supplementary Fig. 1d)^6,11^. We also detected the MERVL-tdTomato clones and observed the lack of chromocenters, a characteristic noted in zygotes and 2-cell-stage embryos (Supplementary Fig. 1e)^12^. Subsequently, we sorted GFP+ and tdTomato+ cells from OG2CT mESCs and performed RNA-seq analysis. The volcano plot and heatmap analysis revealed that tdTomato+ cells upregulated totipotency genes, while GFP+ cells exhibited high expression of pluripotency markers (Supplementary Fig. 2a, b). This confirmed that OG2CT mESCs were suitable for in vitro studies of totipotency. Next, we designed a *piggyBac* sgRNA library comprising 13,000 sgRNAs to ensure comprehensive coverage of the functional genome^13,14^. We co-electroporated a Cas9-expressing plasmid with the sgRNA library plasmid into OG2CT mESCs (Fig. 1a). After 5 days of Neo treatment, we collected and expanded the resistant positive mESCs to a scale of 3 × 10^8^ cells for subsequent screening. To assess the mutant cell pool, we amplified sgRNA cassettes from the genome and performed deep sequencing. This mutant cell pool covered ~89% of sgRNAs, achieving comprehensive genome coverage (Supplementary Fig. 2c). In summary, we successfully constructed a CRISPR/Cas9 mutant library in OG2CT mESCs, providing ample coverage for the screening of totipotency regulatory factors.

Following this, we conducted screening experiments by splitting the mutant cell pool into three parallel experimental groups, resulting in a significant increase in the proportion of 2CLCs (Fig. 1b). We isolated tdTomato+ cells from each group to identify key negative regulators. Genomic DNA was extracted and subjected to deep sequencing, revealing a decrease in sgRNA counts during screening (Supplementary Fig. 2c). Using the MAGeCK algorithm, we assessed sgRNA enrichment and identified several negative regulators associated with the transition to a 2C-like state in mESCs, including *Zmym2* and *Mga*^8,9,15^. Gene ontology and Kyoto Encyclopedia of Genes and Genomes analyses indicated that the enriched genes were associated with cell metabolism, RNA synthesis and the oocyte meiosis pathway, which are crucial for totipotency regulation (Fig. 1c, d)^16–18^.

To identify candidate genes, we utilized a combination of the MAGeCK algorithm and Z-scores to evaluate our screening results. In addition to the highly ranked overlapping genes, we also considered genes associated with specific pathways such as the glycolysis pathway. As a result, we selected 18 hits for CRISPR KO validation based on pathway analysis and ranking. The selected hits included *Thoc1*, *Tcl1*, *Mnt*, *H1foo*, *Zmym2*, *Ddx49*, *Baz1a*, *Ppp2r2c*, *Olfr867*, *Ttc12*, *Mapk10*, *Map2k3*, *Slc26a4*, *Orai1*, *Mdm2*, *Uhrf1*, *Zfp93* and *Eno3* (Fig. 1e).

### Identification of the candidates in totipotency regulation

**Fig. 2:**
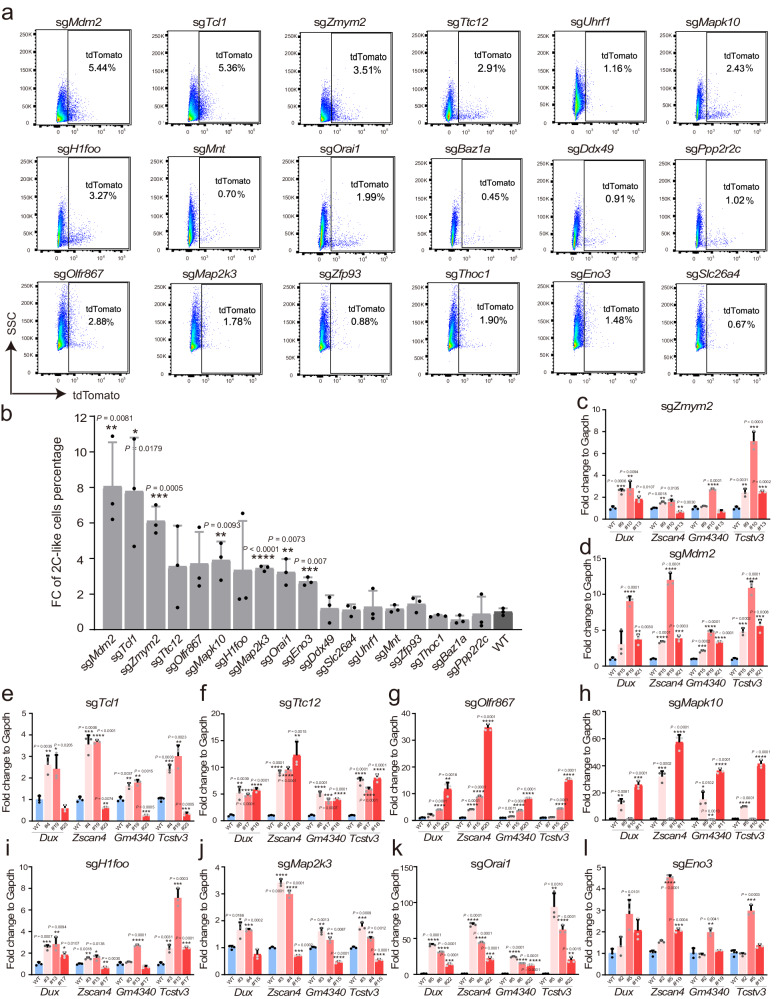
Validation of potential regulators for 2CLCs. **a** FACS analysis demonstrated the population of MERVL-tdTomato reporter upon CRISPR/Cas9 perturbation of 18 candidate genes. **b** The fold change of 2CLCs proportion for 18 candidates compared to WT. n =3 biological replicates. The data were mean ± SD. **c**–**l** RT-PCR results showing relative expression of 2C genes in important candidate gene knockout cell lines. n = 3 biological replicates. The data were mean ± SD. Data are analyzed by Student’s t-test. *P < 0.05, **P < 0.01, ***P < 0.001 and ****P < 0.0001.

To validate whether the candidate genes regulate totipotency, we transfected sgRNAs into mESCs containing the MERVL reporter. While most of these candidates successfully established CRISPR KO cell lines, we encountered significant challenges in generating homozygous *Mdm2* KO cell lines. This observation aligns with existing literature suggesting that the loss of *Mdm2* in mouse embryos can lead to lethality around E3.5^19,20^. As a result, we selected heterozygous *Mdm2* knockdown cell lines for subsequent validation. We quantified the proportion of 2CLCs using FACS analysis in each cell line (Fig. 2a). Ten candidates showed an increase in the proportion of 2CLCs (Fig. 2b). These ten hits included *Mdm2*, *Tcl1*, *Zmym2*, *Ttc12*, *Olfr867*, *Mapk10*, *H1foo*, *Map2k3*, *Orai1* and *Eno3*. Our findings validated the enrichment of potential totipotency regulators identified in our screening. Subsequently, we conducted separate quantitative PCR (qPCR) analyses to measure the expression levels of pluripotency and totipotency genes in the cell lines of the ten hits. After perturbing ten hits, the expression of totipotency markers was upregulated, consistent with the FACS results (Fig. 2c–l). Interestingly, the expression of pluripotency genes was not uniformly downregulated (Supplementary Fig. 3a–j), which may be attributed to the different modes of action of these regulators. Among the ten genes that were further validated, perturbation of *Mdm2*, *Tcl1* and *Zmym2* exhibited the most significant effects in totipotency regulation.

### *Mdm*2 is required for totipotency exit

**Fig. 3:**
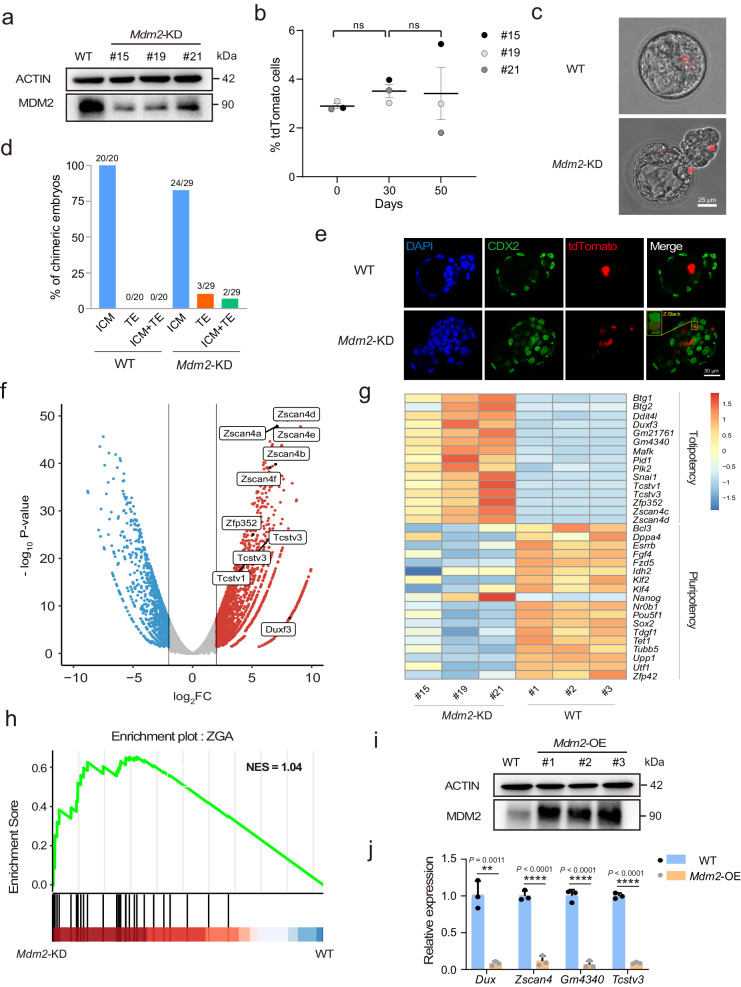
*Mdm2* inhibited the transcription of 2C genes in mESCs. **a** Western blot showed the result of *Mdm2* expression in *Mdm2* KD mESCs. The experiment was repeated three times and gave similar results. **b** The proportion of MERVL-tdTomato fluctuated over time in *Mdm2* KD mESCs. **c** Microscope pictures (10×) of chimeric embryos injected with tdTomato-labeled WT and *Mdm2* KD mESCs. **d** Summary of the proportion of E4.5 chimeric embryos from injections of WT and *Mdm2* KD mESCs into 8-cell stage embryos. **e** The results of immunofluorescence showed the developmental progression of tdTomato-labeled mESCs into the ICM and TE. **f** Volcano plot depicting the differentially expressed genes between in *Mdm2* KD and WT mESCs. Red dots represented the genes of log2 FC > 2 and blue dots represented the genes of log2 FC < −2. We specifically labeled 2C genes. n = 3 biological replicates per group. **g** Heatmap analysis of 2C genes and pluripotency genes in *Mdm2* KD and WT mESCs. n = 3 biological replicates per group. **h** GSEA analysis indicated upregulation of ZGA in *Mdm2* KD mESCs compared with WT mESCs. **i** Western blot showed the result of *Mdm2* overexpressing. The experiment was repeated three times and obtained similar results. **j** The relative expression of 2C genes in *Mdm2* OE mESCs by RT-PCR. n = 3 biological replicates. Data are analyzed by Student’s t-test. The data were mean ± SD. **P < 0.01, and ****P < 0.0001.

We focused on the function of *Mdm2* as a negative regulator of 2C genes. When we used CRISPR/Cas9 to generate *Mdm2*-deficient mESCs, the cell morphology did not show significant differences compared to the wild type (Supplementary Fig. 4a). Western blot analysis revealed a significant decrease in *Mdm2* expression levels in three different clones (Fig. 3a). After perturbing *Mdm2*, we observed a further downregulation of several pluripotency genes (Supplementary Fig. 4b). We cultured *Mdm2* KD mESCs for an extended period to detect whether the proportion of 2CLCs could be maintained. FACS analysis revealed that mESCs maintained a higher proportion of 2CLCs for up to 50 days and 17 passages, indicating that the effect of *Mdm2* deficiency on totipotency was stable (Fig. 3b). We also conducted a chimera assay to assess the developmental potential of *Mdm2* KD mESCs. Subsequently, we evaluated the differentiation capabilities of these tdTomato-positive cells. Our findings revealed that *Mdm2* KD mESCs demonstrated a notable capacity to integrate into the extra-embryonic trophectoderm in ~17% of chimeric blastocysts. In contrast, WT mESCs were exclusively observed to contribute to the inner cell mass in all blastocysts (Fig. 3c–e). To further demonstrate the capacity of *Mdm2* knockdown to induce 2CLCs, we performed RNA-seq. We found that the expression levels of totipotency genes, including *Dux* and the *Zscan4* cluster genes, increased, while the expression of several pluripotency genes was downregulated, consistent with the quantitative results following *Mdm2* knockdown (Figs. 2d and 3f, g). Meanwhile, RNA-seq analysis showed the upregulation of the ZGA gene set and the downregulation of the spliceosome gene set, in line with previously published data (Fig. 3h, Supplementary Fig. 4c)^21^. A previous study reported that the transition of mESCs to 2CLCs occurs in two steps: first, pluripotent genes were downregulated, and then totipotency genes were upregulated^10^. Therefore, we investigated at which step *Mdm2* impeded the transition to 2CLCs. To test this, we identified significantly different genes in sg*Mdm2* mESCs (|FC| > 2 and P value < 0.05). We found that 43 of the activated genes after *Mdm2* deficiency belonged to the 2C-upregulated gene set, and 46 of the repressed genes after *Mdm2* deficiency belonged to the 2C-downregulated gene set, suggesting that there was no significant difference in the number of activated or repressed genes (Supplementary Fig. 4d). However, the expression changes were more pronounced in 2C-upregulated genes and 2C-downregulated genes after *Mdm2* deficiency, indicating that *Mdm2* may preferentially regulate the proportion of 2CLCs by targeting 2C-upregulated genes (Supplementary Fig. 4e). Furthermore, we successfully constructed a flag-tagged *Mdm2* into mESCs (Fig. 3i). The overexpression of *Mdm2* led to a significant downregulation of totipotency genes (Fig. 3j). These cumulative findings strongly supported the role of *Mdm2* as a negative regulator of 2CLCs and as an impediment to the totipotency state.

### *Mdm2* deficiency causes cell cycle arrest at G1 phase

**Fig. 4:**
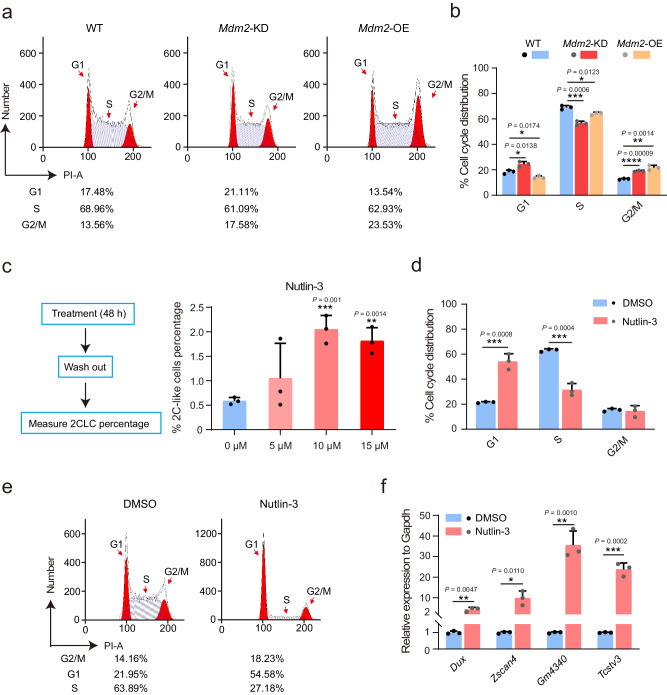
*Mdm2* deficiency caused cell cycle arrest at G1 phase to regulate 2C-like state. **a**, **b** FACS analysis showed the distribution of cell cycle phases on *Mdm2* KD, *Mdm2* OE and WT mESCs. n = 3 biological replicates. **c** Various concentrations of Nutlin-3 induce changes in the percentage of 2CLCs. n = 3 biological replicates per group. **d**, **e** FACS showed cell cycle phases distribution in mESCs treated with 10 μM Nutlin-3. n = 3 biological replicates per group. **f** The relative expression of 2C genes in mESCs treated with 10 μM Nutlin-3. n = 3 biological replicates. Data are analyzed by Student’s t-test. The data were mean ± SD. *P < 0.05, **P < 0.01, ***P < 0.001 and ****P < 0.0001.

Gene set enrichment analysis (GSEA) analysis showed a notable reduction in the cell cycle gene set in *Mdm2* KD mESCs when compared to WT mESCs. This reduction is consistent with the downregulation observed in 2CLCs versus WT mESCs (Supplementary Fig. 5a, b). Furthermore, in line with the GSEA results, *Mdm2* knockdown led to decreased cell proliferation compared to mESCs (Supplementary Fig. 5c). To comprehend the role of *Mdm2* in cell cycle regulation, we analyzed the proportions of cells in *Mdm2* KD, *Mdm2* OE and WT mESCs. We observed significant changes in the G1 phase (Fig. 4a, b). The reduction of *Mdm2* expression resulted in an increased proportion of mESCs arrested in the G1 phase, promoting the emergence of a 2C-like state^18^. Conversely, the overexpression of *Mdm2* led to a shortened G1 phase, a characteristic feature of pluripotency^22^. Next, we sought to confirm the functional role of *Mdm2* by subjecting mESCs to Nutlin-3 treatment, a specific inhibitor of *Mdm2*^23^. Initially, we assessed the impact of different concentrations of Nutlin-3 and found that 10 μM Nutlin-3 significantly induced a substantial proportion of 2CLCs (Fig. 4c). Notably, exposure to Nutlin-3 resulted in a pronounced cell cycle arrest at the G1 phase (Fig. 4d, e). Consistent with these observations, we observed a significant upregulation of 2C genes following Nutlin-3 treatment (Fig. 4f). In summary, the cumulative data strongly suggested that *Mdm2* deficiency had the potential to initiate totipotency activation by inducing cell cycle arrest at the G1 phase^18,24,25^.

### *Mdm2* affects H3K27me3 modification at *Dux* locus

**Fig. 5:**
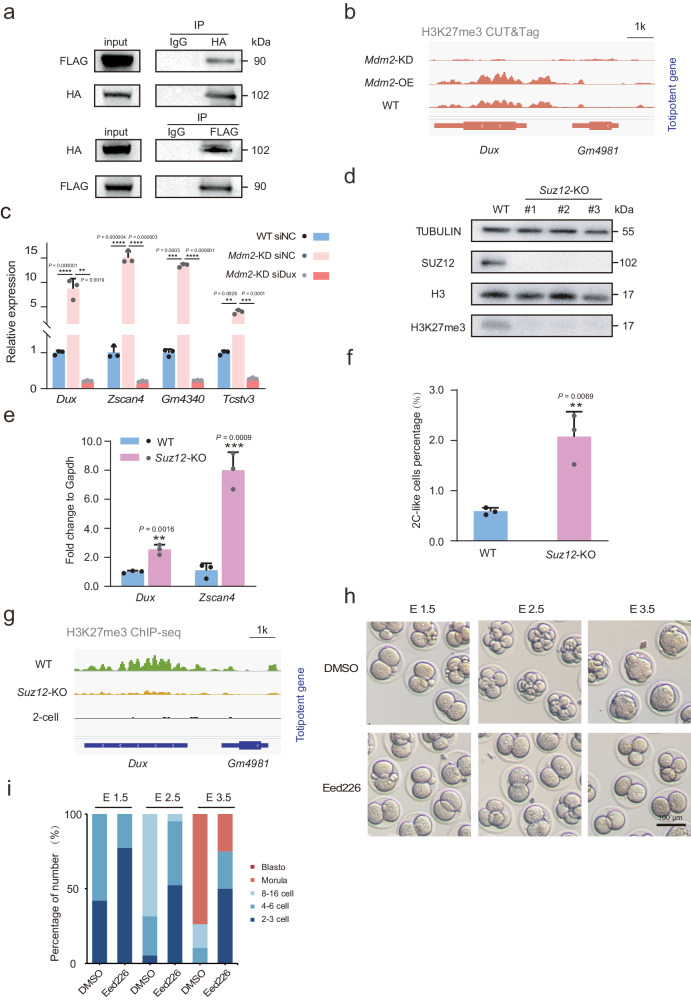
*Mdm2* enhanced H3K27me3 modification at *Dux* in mESCs. **a** Western blotting of MDM2 and SUZ12 for Co-IP results. **b** IGV view of H3K27me3 modification at the *Dux* locus in *Mdm2* KD, *Mdm2* OE and WT mESCs. **c** The relative expression of 2C genes in WT and *Mdm2* KD mESCs subjected to either negative control or *Dux* siRNA treatment. n = 3 biological replicates. **d** Western blot showing the SUZ12 and H3K27me3 expression level in WT and *Suz12* KO mESCs. **e** The percentage of 2CLCs in *Suz12* KO mESCs. n = 3 biological replicates. **f** The relative expression of 2C genes in *Suz12* KO mESCs. n = 3 biological replicates. **g** IGV view of H3K27me3 modification at the *Dux* locus in *Suz12* KO, WT mESCs and 2C embryo. **h**, **i** Representative images and developmental percentages of embryos at different phases following treatment with DMSO or EED226. n = 3 biological replicates. Scale bar, 100 μm. Data are analyzed by Student’s t-test. The data were mean ± SD. **P < 0.01, ***P < 0.001 and ****P < 0.0001.

*Dux* functions as a master activator of the 2C-like state. Initially, we sought to determine whether *Mdm2*, as a transcription factor, would bind to *Dux*. Using FLAG CUT&Tag in *Mdm2* OE mESCs, we found no evidence of *Mdm2* occupancy at the *Dux* locus or other 2C genes such as *Zscan4*. This led us to hypothesize that *Mdm2* might exert its regulatory role in the totipotency state through alternative nuclear mechanisms. Recent studies have reported an interaction between *Mdm2* and the polycomb repressor complex 2 (PRC2), which enhances the trimethylation of histone H3 lysine 27 (H3K27me3)^26,27^. To investigate the interaction between MDM2 and PRC2, we conducted co-immunoprecipitation (Co-IP) assays in 293T cells. Our results indicated that the SUZ12 antibody, a core component of PRC2, selectively precipitated MDM2. Reciprocal Co-IP experiments further demonstrated that MDM2 was able to co-precipitate SUZ12 as well (Fig. 5a). To investigate this further, we assessed the levels of H3K27me3 modification in *Mdm2* KD, *Mdm2* OE and WT mESCs. Our observations demonstrated that *Mdm2* perturbation reduced H3K27me3 modification levels, particularly at the *Dux* locus, while *Mdm2* overexpression increased H3K27me3 levels at the *Dux* locus (Fig. 5b). Furthermore, upon knocking down *Dux* using siRNA in the *Mdm2* KD mESCs, we found that the upregulation of totipotency genes was abolished (Fig. 5c). This indicated that *Mdm2* had the potential to enhance H3K27me3 modification by influencing *Dux* expression, thereby influencing the regulation of totipotency. Western blot analysis unveiled no substantial alteration in the global H3K27me3 modification levels in both *Mdm2* KD and *Mdm2* OE mESCs (Supplementary Fig. 6a). We further scrutinized H3K27me3 modifications within the promoter region, and the outcomes indicated a lack of statistical significance across the specified mESCs (Supplementary Fig. 6b). These observations collectively suggested that *Mdm2* selectively inhibited *Dux* expression through its H3K27me3-mediated regulatory mechanism.

Some literature has described the H3K27me3 profile in mouse pre-implantation embryos, revealing that the H3K27me3 features in the 2CLCs resemble those of mESCs more closely than 2C embryos. This suggests that only a few critical genes acquire the 2C embryo-specific epigenetic signatures during the transition to the 2C-like state^28,29^. Given these observations, we investigated whether H3K27me3 modification levels influenced the 2C-like state. The PRC2 complex is responsible for H3K27 methylation and the production of H3K27me3 modification^30^. In mouse early embryos, we observed the expression of core PRC2 subunits, namely EZH2, SUZ12 and EED (Supplementary Fig. 6c)^31^. Notably, *Suz12* exhibited strong expression in 2-cell embryos and maintained high expression in the inner cell mass (ICM). Therefore, we focused on the function of *Suz12* and found that *Suz12* knockout resulted in the erasure of overall H3K27me3 in mESCs (Fig. 5d). Depletion of *Suz12* also led to an increased proportion of 2CLCs and elevated expression levels of 2C genes (Fig. 5e, f). To gain further insight about *Suz12*, we reanalyzed H3K27me3 epigenomic datasets in *Suz12* knockout mESCs^32^. We observed a decrease in H3K27me3 intensity specifically at the *Dux* locus in *Suz12* knockout compared to WT mESCs, resulting in a profile more similar to 2C embryos than mESCs (Fig. 5g)^28,29^. Conversely, H3K27me3 binding to other 2C key genes, such as *Zscan4c* and *Zscan4d*, remained unaffected following *Suz12* knockout (Supplementary Fig. 6d). Furthermore, upon evaluating the H3K27me3 profiles of pluripotent genes, we observed an augmentation rather than a reduction in H3K27me3 modification. This heightened modification could conceivably impede pluripotent genes, thereby facilitating the emergence of the 2C-like state (Supplementary Fig. 6e). To further validate the contribution of H3K27me3 modification during embryonic development, we treated zygotes with EED226, a specific inhibitor of the PRC2 complex^33^. This treatment resulted in a delay in zygote development in vitro (Fig. 5h, i). These results underscore the significance of H3K27me3 in regulating totipotency and *Mdm2* could dictate cell fate by H3K27me3 modification at *Dux*.

### *Mdm2* deficiency influences embryonic development

**Fig. 6:**
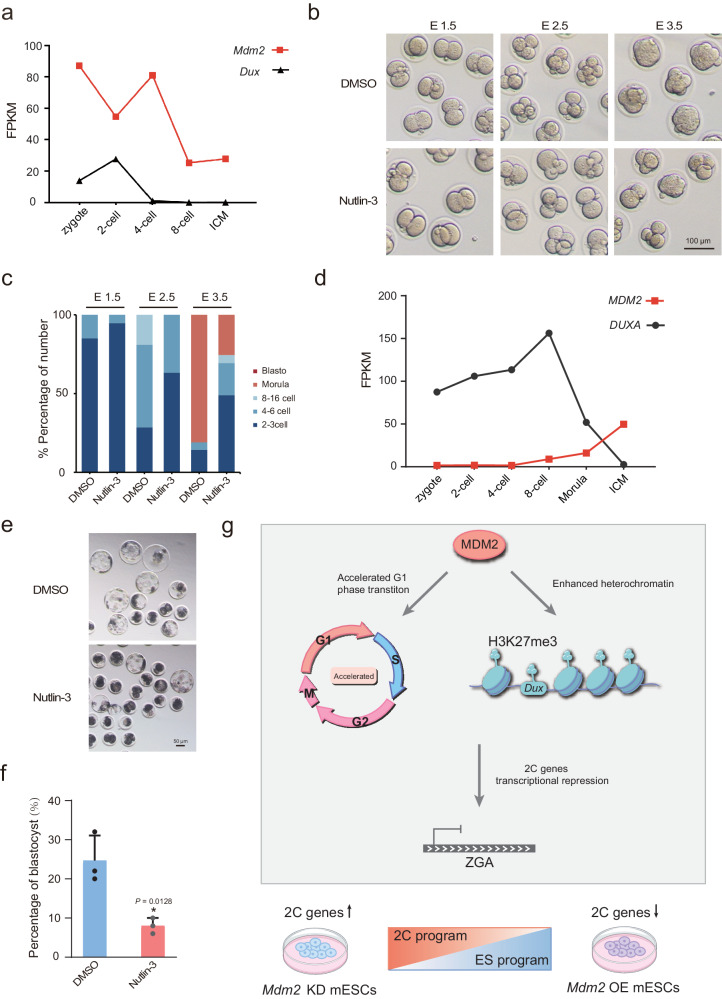
*Mdm2* regulated the embryonic development. **a** *Mdm2* and *Dux* expression level according to RNA-seq during mouse pre-implantation embryos. **b**, **c** The representative images and developmental percentages of mouse embryos at different phases following treatment with DMSO or Nutlin-3. n = 3 biological replicates. Scale bar, 100 μm. **d** MDM2 and DUXA expression level according to RNA-seq during in pig pre-implantation embryos. **e** The microscopy images showing the blastocyst formation in DMSO and Nutlin-3 in groups. Scale bar, 50 μm. **f** The bar plot displaying the blastocyst formation rates in DMSO and Nutlin-3 in groups. n = 3 biological replicates. **g** The models depicting the *Mdm2*-mediated regulatory mechanisms of the totipotency state and ZGA. Data are analyzed by Student’s t-test. The data were mean ± SD. *P < 0.05.

*Dux* exhibits elevated expression levels in 2C embryos, corresponding to the early developmental phases characterized by active ZGA, and subsequently, its expression is downregulated and ultimately silenced^34^. We assessed the expression levels of *Mdm2* in pre-implantation embryos^31^ and observed a decrease in *Mdm2* expression during the 2-cell stage, followed by an increase at the 4-cell stage, in contrast to the expression pattern of *Dux* (Fig. 6a). This suggested that the upregulation of *Mdm2* was required for the termination of ZGA and the exit from the totipotent state. To gain deeper insights into the role of *Mdm2* in early embryo development, we treated single-cell mouse fertilized embryos with Nutlin-3. Remarkably, Nutlin-3 had a significant adverse effect on the development of early mouse embryos, resulting in a higher number of embryos arrested at the 2-cell stage (Fig. 6b, c). This observation underscored the essential role of *Mdm2* in the process of pre-implantation embryo development. Given the critical involvement of *Mdm2* in the regulation of ZGA and subsequent embryonic development in mouse embryos, we sought to explore whether a similar mechanism operates in porcine embryos. Porcine ZGA occurred during the four- to eight-cell stages^35^. Our investigation into the expression profiles of MDM2 and the porcine double homeobox gene, DUXA, revealed expression patterns closely resembling those observed in mice (Fig. 6d)^36^. As expected, the group treated with Nutlin-3 exhibited a reduced blastocyst development rate, and a higher proportion of embryos were arrested at the early embryos state (Fig. 6e, f). In conclusion, the mechanism of *Mdm2*-mediated regulation appeared to be conserved between mice and porcine, highlighting its critical significance in mammalian embryo development.

## Discussion

Here, we performed a genome-wide CRISPR KO screen to elucidate the precise regulation of totipotency and pluripotency by employing totipotent-like models in mESCs. In contrast to previous CRISPR KO screens, our screen did not rely on totipotency activators for completion^10^. This method presented challenges, mainly due to technical difficulties stemming from the lower proportion of 2CLCs. To address these challenges, we expanded the mutant pool to encompass 3 × 10^8^ cells. Our screen achieved a successful identification of authentic hits, which are well-recognized for their involvement in orchestrating the transition of totipotency and pluripotency, such as *Zmym2*, *Uhrf1*, *Mga*^10,15,37^. We carefully selected 18 candidates for validation, and nearly 50% of the candidates were perturbed to effectively increase the proportion of 2CLCs, thereby uncovering several factors previously unassociated with the 2C-like state. These candidates arise from disparate pathways; however, they may exhibit interconnections. We have observed that Nutlin-3, an inhibitor targeting *Mdm2*, possesses the ability to reduce *Tcl1* expression in primary B chronic lymphocytic leukemia cells^38^. *Tcl1*, recognized as a crucial pluripotency factor, was recently reported to facilitate metabolic shifts implicated in the transition to the totipotent state^16,39^. Our screening has unveiled that, besides *Tcl1*’s involvement in energy transition promotion, *Eno3*, an enzyme associated with glycolysis, acts as a regulator of totipotency. Additionally, our findings suggested that these candidates played a role in modulating *Dux* expression, potentially positioning them as regulators acting upstream of *Dux*. Therefore, a more in-depth analysis of these newly identified candidates promises to provide profound insights into the underlying network of totipotency.

In our validation studies, we noted a significantly higher proportion of 2CLCs, with a remarkable tenfold increase observed following the perturbation of *Mdm2*, *Tcl1* and *Zmym2*. Furthermore, the perturbation of these factors exerted a notable influence on the expression of genes associated with both totipotency and pluripotency. *Zmym2* had been previously identified, and its knockout was found to result in compromised transitions from totipotency to pluripotency^15^. *Zmym2* facilitated the recruitment of HDAC-containing complexes, leading to their binding to MERVL and subsequent repression of MERVL expression. Therefore, further investigations are necessary to study other unreported factors.

*Mdm2* is traditionally known as a primary inhibitor of P53 and many studies develop *Mdm2* inhibitors to activate P53 for cancer treatment^40^. Recent studies have shown that *Mdm2* individual function independent of P53 and its knockout in P53 deficiency also promotes cancer cell death^27,41^. Obviously, *Mdm2* and P53 are highly valued in cancer therapy. Recently, a report has elucidated that P53 serves as a definitive activator of *Dux* to induce 2CLCs^42^. This discovery suggests that *Mdm2* may regulate 2CLCs through its interaction with P53. However, the exact role of *Mdm2* in this scenario has not been fully understood until now.

Our study observed a contrasting pattern in the expression levels of *Mdm2* during the 2-cell and 4-cell developmental stages, which differed from that of *Dux*, which is 2C activators. This discrepancy suggested the necessity of *Mdm2* emergence for the transition out of totipotency. *Mdm2* played a dual role in cellular processes. First, it exhibited the capacity to modulate the cell cycle, thereby influencing cell fate determination. *Mdm2* induced a speeding up of the G1 phase, leading to the establishment of the pluripotent state^18,43,44^. The control of the cell cycle is crucial for tightly regulating cell numbers in early embryo development and it also plays a fundamental role in governing the balance between self-renewal and differentiation in stem cells. Second, *Mdm2* facilitated the augmentation of H3K27me3 modifications, particularly at the *Dux* locus to facilitating the exit of totipotency. This histone modification, H3K27me3, is recognized for its role in gene silencing^45,46^. And *Mdm2* KO embryos display post-implantation lethality at E3.5^19,20^. These data signify that *Mdm2* activity primarily played a vital role in early embryos and is essential for ensuring normal embryo development.

In summary, our findings support a novel model that underscores the pivotal role of *Mdm2* in orchestrating the precise transition of totipotency to pluripotency while preventing its protracted totipotent state. This regulatory function is paramount for the successful advancement of pre-implantation development (Fig. 6g). Notably, our observations in pig embryos revealed that Nutlin-3, an inhibitor of *Mdm2*, treatment could impede pre-implantation development, suggesting a conserved mechanism of *Mdm2*-mediated ZGA regulation across mammalian species. Furthermore, our screening efforts unveiled a spectrum of negative factors implicated in totipotency and the 2CLCs, thus contributing to illuminate an expanded regulatory network.

## Materials and methods

### Mice

All mice were housed at the China Agricultural University Laboratory Animals Resource Center, where they were subjected to a standard light–dark cycle of 12 h each, and maintained at a temperature of 20–22 °C. All animal experiments were conducted in Sen Wu’s laboratory and were approved by the Institutional Animal Care and Use Committee of China Agricultural University (Approval Number: SKLAB-2012-11). We have complied with all relevant ethical regulations for animal use. The 5 weeks old female ICR mice were procured from Beijing Vital River Laboratory Animal Technology.

### Cell culture

The OG cell lines were maintained on feeder cells using a basic serum/LIF medium. This medium comprised DMEM (Gibco, 10829018), 15% FBS (Gibco, 10099), 1% penicillin/streptomycin (Gibco, 15104122), 1% non-essential amino acids (Gibco, 11140050), 1% GlutaMAX (Gibco, 35050079), 10^6^ units/L of mouse LIF (Millipore, ESG1106) and 100 mM β-mercaptoethanol (Gibco, 15104122). The feeder cells were treated with mitomycin C (Amresco, MJ594) to inhibit their growth and were cultured in DMEM (Gibco, 11960) supplemented with 10% FBS (Gibco, 10099), 1% penicillin/streptomycin (Gibco, 15104122), 1% non-essential amino acids (Gibco, 11140050) and 1% sodium pyruvate (Gibco, 15104122). Other small molecules mentioned in our article were procured from Selleck and added individually to the basal serum/LIF medium at varying concentrations. Subculturing of all cell lines was performed every 2–3 days at a ratio ranging from 1:6 to 1:10 using Tryple (Gibco, 12605028). To establish the MERVL-tdTomato reporter cell lines, the OG cell lines underwent transfection via electroporation and were subsequently selected using 1 µg/ml puromycin for 7 days. Clones were then isolated and confirmed through polymerase chain reaction (PCR) analysis. All cell lines were maintained in a humidified incubator at 37 °C with 5% CO_2_.

### Genome-wide CRISPR KO screens

The CRISPR-Cas9 DNA library contained 130,209 sgRNAs about 20,611 genes constructed by our laboratory. To obtain the mutant cell library, we transfected 10^8^ OG cells with the MERVL reporter by electroporation (2B Nucleofector System, Lonza). After 24 h transfection, the cells were selected with 350 ng/µl G418 (InvivoGen, ant-gn-5) for 7 days. We expanded these cells to 3 × 10^8^ for primary screen. We collected 2C-positive cells using FACS and amplified them for consecutive screen. Each round of screening was repeated three times. Medium needed to be changed every day.

Genomic DNA were extracted from 10^8^ cells of the mutant cell pool and 5–10 × 10^6^ cells of 2C-postive from the consecutive screen. The integrated sgRNA sequences were amplified by PCR using Gotaq DNA Polymerase (Mei5bio, MF002) with the left primer (5′-AATGATACGGCGACCACCGAGATCTACACTCTTTCCCTACACGACGCTCTTCCGATCTNNNNNNNNTGAAAGTATTTCGATTTCTTGG-3′) and the right primer (5′- CAAGCAGAAGACGGCATACGAGATNNNNNNNNGTGACTGGAGTTCAGACGTGTGCTCTTCCGATCTTGTTGATAACGGACTAGCCTTATT-3′). Then PCR products were purified and sequenced by Illumina HiSeq TM4000. We used MAGeCK package to analyze our screening results and took Z-score into consideration. Ggplot2 was used to summarize these results.

### Generation of CRISPR KO cells

To generate CRISPR KO cells, we designed two sgRNAs for each target gene and cloned them into the pSg6 Plasmid. Subsequently, we transfected 10^6^ mESCs using the Lonza 2B Nucleofector System. Twenty-four hours after transfection, cells were subjected to selection with 350 ng/µl G418 for a duration of 7 days. Following selection, individual clones were isolated into a 48-well plate, and their targeted regions were verified. Detailed sequences of the sgRNAs can be found in Table S1.

### Overexpression of MDM2 in mESCs

Total RNA was extracted to amplify the CDS of *Mdm2* by PCR. Subsequently, the amplified fragment was cloned into the PB vector under the control of the EF1α promoter. A total of 4 µg of the vector was transfected into 10^6^ mESCs using the Lonza 2B Nucleofector System. Following transfection, mESCs were cultured for 24 h and then subjected to selection with 350 ng/µl G418 for a period of 7 days. Clones were subsequently isolated and expanded in a 48-well plate to allow for the extraction of RNA and protein.

### RNA extraction and qPCR

Total RNA was extracted from cells using the RaPure Total RNA Kits (Magene, R4011) following the manufacturer’s protocol. Subsequently, 1 µg of RNA was reverse transcribed into cDNA using the ABScript III RT Master Mix (Abclonal, RK20429). qPCR was conducted using the 2x RealStar Green Power Mix (Genestar, A311-10) on a Roche PCR machine. The relative quantification of each gene was achieved by normalizing to *Gapdh*. A complete list of primers utilized for qPCR can be found in Table S2.

### RNA sequencing and bioinformatics analysis

Total RNA was purified using magnetic beads with Oligo (dT) to selectively isolate mRNA. RNA-seq libraries were subsequently constructed and assessed using a combination of TIANGEN Biotech and the Agilent 2100 BioAnalyzer. The sequencing process generated 150 bp paired-end reads using PE150 on an Illumina platform. To analyze the data, clean reads were mapped to the Mus musculus genome using HISAT2. Read counts were quantified using HTSeq-count (v0.6.0) and then normalized to obtain the Fragments Per Kilobase of transcript sequence per Millions base pairs sequenced (FPKM) values. Differentially expressed genes (DEGs) were identified using edgeR with criteria including an absolute log2 (fold change) > 2 and a p value < 0.05. Subsequently, the datasets were further analyzed using the R programming language.

### Flow cytometry

Cell sorting and analysis were carried out using the FACS CaliburTM flow cytometer (BD, San Jose, CA, USA). Data visualization was conducted using FlowJo software version 10. For our gating strategy, we employed wild-type (WT) mESCs with the MERVL reporter.

### Co-immunoprecipitation

Collect 293T cells cultured in 10 cm culture dishes using 1 mL of IP lysis buffer (Beyotime, P0037), and transfer them to 1.5 mL centrifuge tubes. Let them lyse on ice for 30 min. After centrifugation at maximum speed (4 °C, 21,100 g, 10 min), collect the supernatant for subsequent Co-IP experiments. Supernatants were incubated with Pierce™ beads (Thermo Scientific™, 88802) for 6–8 h at 4 °C with rotation. Antibodies used include FLAG (Sigma, F1804, 1:200 mouse) and HA (Beyotime, AH158, 1:200 mouse), IgG (Beyotime, A7028, 1:200 mouse). The protein solution collected was denatured at 95 °C for 10 min using 10% SDS–PAGE for western blot analysis.

### Western blotting

The cells were lysed using IP lysis buffer supplemented with 100× PMSF and incubated on ice for 30 min. The supernatant was obtained by centrifuging at 4 °C and 20,000 × *g* for 15 min. To determine protein concentration, we utilized the BCA protein assay kit (Beyotime, P0012) according to the manufacturer’s instructions. Equal amounts of protein were denatured using 10% SDS–PAGE. For western blotting, the following primary antibodies were used: MDM2 (Abcam, ab259265, 1:1000 rabbit), SUZ12 (Cell Signal Technology, 3737, 1:1000 rabbit), ACTIN (Beyotime, AA128, 1:1000 mouse), TUBULIN (Beyotime, AF2835, 1:1000 mouse), HA (Beyotime, AH158, 1:1000 mouse), FLAG (Sigma, F1804, 1:1000 mouse), H3 (Elabscience, E-AB-22003, 1:1000 mouse) and H3K27me3 (Sigma, 07-449, 1:10,000 rabbit).

### Immunofluorescence

Embryos were subjected to fixation with 4% paraformaldehyde for a duration of 30 min and subsequent permeabilization using 0.5% Triton X-100 for 30 min at room temperature. Blocking of embryos was treated with 1% BSA in PBS supplemented with 0.1% Tween 20, lasting for 60 min at room temperature. Incubation with CDX2 primary antibodies (Biogenex, MU392A, 1:200 mouse) occurred overnight at 4 °C, followed by incubation with secondary antibodies (Invitrogen, A32723, 1:500) for 1 h at room temperature. Lastly, the nuclei of the embryos were stained with DAPI (Beyotime, P0131), and images were acquired using the fluorescence microscope.

### Cell cycle analysis

Cells were fixed by incubating with 70% ethanol at −20 °C overnight. The next day, the fixed cells were centrifuged at 4 °C, 300 × *g*, and washed once with PBS. Afterward, RNase A treatment was performed at 37 °C for 30 min. Finally, the cells were stained with propidium iodide (PI) at 4 °C for 30 min. Cell cycle analysis was performed using a BD flow cytometer, and the data were analyzed with Modfit LT software to determine the distribution of cells across different phases of the cell cycle.

### Nutlin-3 treatment

To inhibit the expression of *Mdm2* in pre-ZGA embryos, zygotes were cultured in KSOM or HM medium supplemented with Nutlin-3 (Selleck, S1061) at a final concentration of 5 μM. DMSO was included as a negative control. The embryos were cultured at 37 °C and monitored daily until they reached the blastocyst stage.

### CUT&Tag

CUT&Tag assay was conducted using the NovoNGS CUT&Tag 3.0 High-Sensitivity Kit (Novoprotein, N259). Approximately 1 × 10^5^ mESCs were incubated with 10 μL of Binding ConA beads. Primary antibodies targeting FLAG (Sigma, F1804, 1:50 mouse) and H3K27me3 (Sigma, 07-449, 1:100, rabbit) were incubated overnight at 4 °C. Subsequently, secondary antibodies were added, and the mixture was incubated at room temperature for 1 h. Following this, the samples were treated with 1 μL of Transposome and incubated at room temperature for 1 h. DNA was then collected for subsequent PCR analysis. The libraries were amplified and subjected to sequencing using the Illumina NovaSeq PE150 platform following the manufacturer’s instructions.

### ChIP-seq and CUT&Tag data analysis

The raw data underwent quality filtering using Trimmomatic to obtain clean data. These clean data were then aligned to the mm10 genome using Bowtie2. For the identification of peaks, we utilized MACS2. Heatmaps were generated using Deeptools, and ChIPseeker was employed to annotate the promoters.

### Public datasets reanalyzed

We conducted a re-analysis of publicly available datasets. H3K27me3 ChIP-seq data for mESCs and SUZ12 KO mESCs were obtained from GSE103685. Additionally, H3K27me3 modification data for 2C embryos were acquired from GSE73952, and RNA-seq data for mouse and pig pre-implantation embryos were retrieved from GSE71434 and GSE163709.

### Statistics and reproducibility

The statistical differences were analyzed by the Student’s t-test when two independent groups were compared. Data are displayed in a bar graph with error bars representing the mean ± SD and individual sample points shown. GraphPad Prism was used for the statistical analysis of data. *P < 0.05, **P < 0.01, ***P < 0.001 and ****P < 0.0001. Three independent biological replicates were included and the figure legends specify the sample sizes.

### Reporting summary

Detailed information on the research design can be found in the Nature Portfolio Reporting Summary associated with this article.

### Supplementary information

Supplementary Material

Description of Additional Supplementary Files

Supplementary Data 1

Reporting-summary

## Data Availability

Additional data are accessible from the corresponding authors. Supplementary Fig. S8 contains uncropped blot images. Source data underlying the article’s graphs and Supplementary Information can be found in Supplementary Data 1. The RNA-seq and Cut Tag data produced in this study are available at GEO with accession numbers GSE269168 and GSE269169.
