# Supplementary material for: A CRISPR/Cas9 screen in embryonic stem cells reveals that Mdm2 regulates totipotency exit
Source: Commun Biol. 2024 Jul 3;7:809. doi: 10.1038/s42003-024-06507-9 (PMC11222520; doi:10.1038/s42003-024-06507-9)
Supplement: Supplementary file 1 — Supplementary Material [file 42003_2024_6507_MOESM1_ESM.pdf]

Supplementary Figure 1. The establishment and identity of 2CLCs reporter

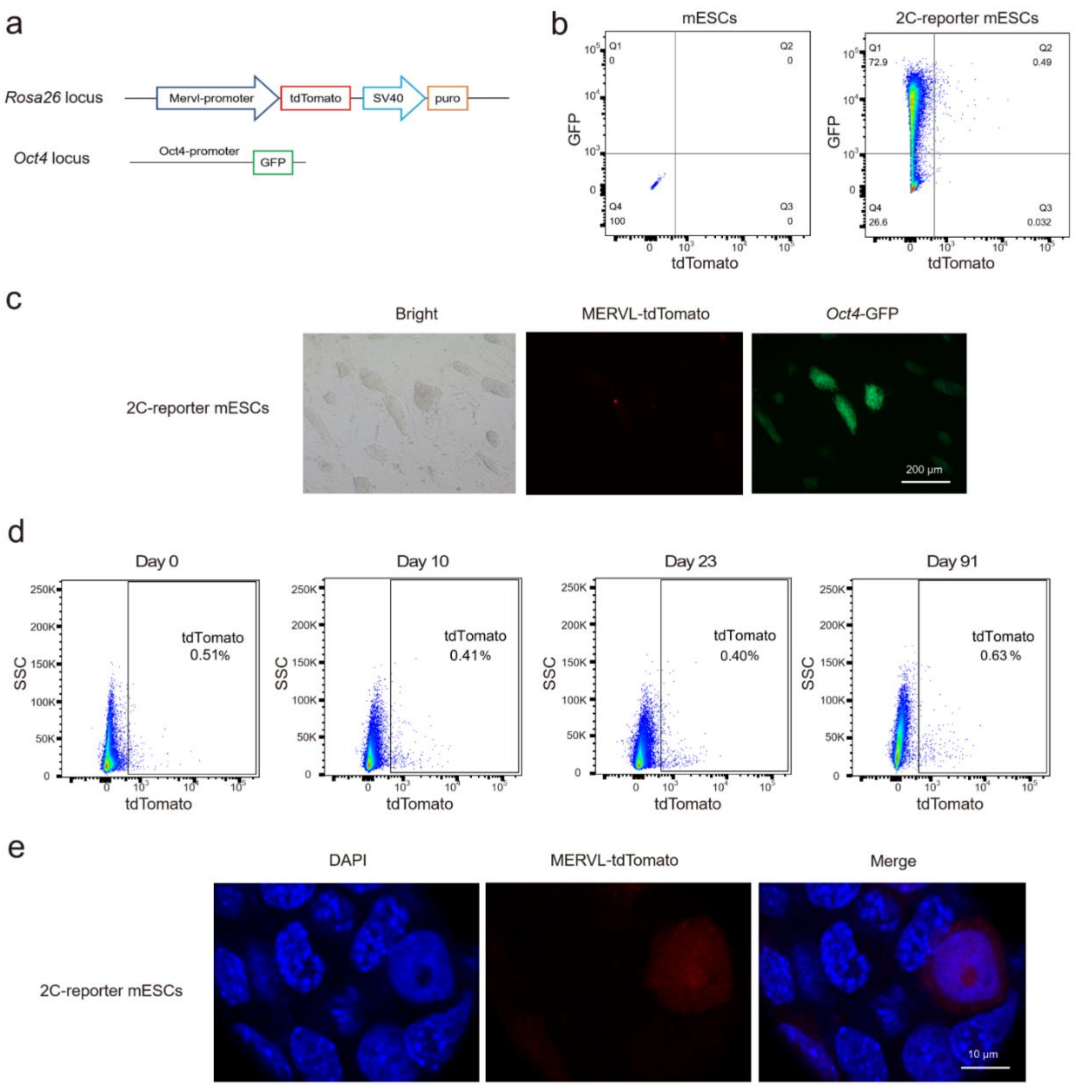

a. A construction strategy of the reporter cell line including the OCT4-GFP and MERVL-tdTomato.

b. FACS quantifying the tdTomato and GFP percentage of the reporter cell line.

c. Microscope pictures (10x) of the reporter cell line.

d. FACS showed the percentage of 2CLCs fluctuates with time in WT mESCs.

e. Representative images of immunostaining performed in OG2CT.

Supplementary Figure 2. Analysis of the mutant cell library and screening results

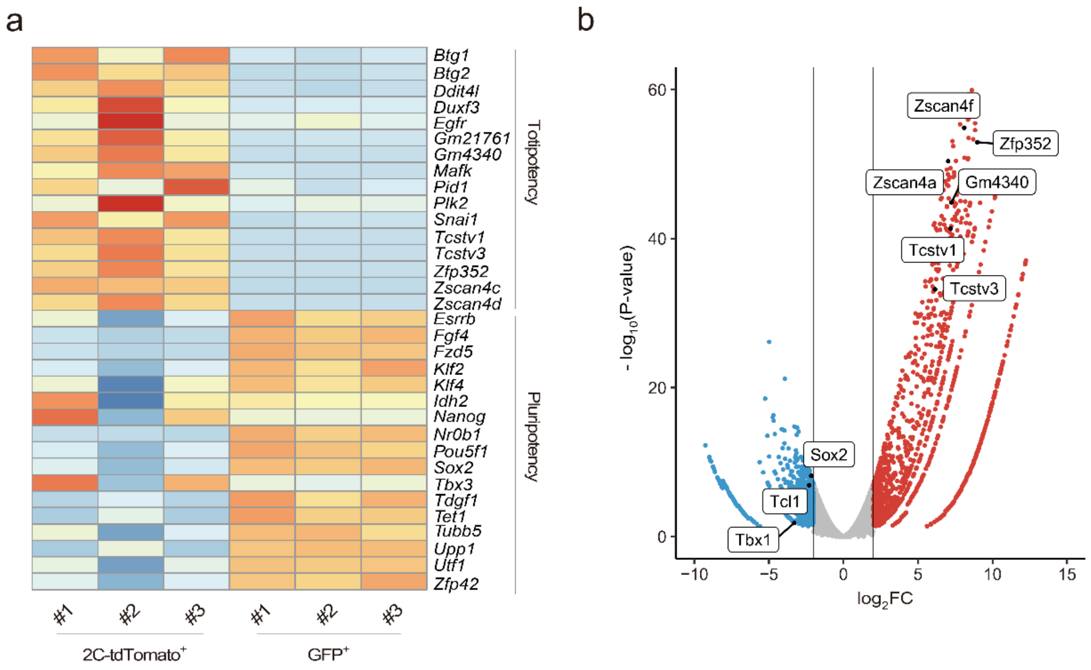

c

	Mutant cell pool		Screen	
	Reads	sgRNA Counts	Reads	sgRNA Counts
Rep1	8,643,534	115,794	9,939,490	9,714
Rep2	8,478,764	116,560	13,955,146	12,509
Rep3	5,213,020	113,264	4,833,161	7,961

a. Heatmap analysis of 2C genes and pluripotency genes in 2CLCs. n = 3 biological replicates per group.

b. Volcano plot of differentially expressed genes between 2CLCs and mESCs. Red dots represented the genes of log2FC> 2 and blue dots represented the genes of log2FC< -2. We specifically labeled 2C genes and pluripotency key genes. n = 3 biological replicates per group.

c. Summary of Illumina read counts and sgRNA abundance for individual samples is presented.

Supplementary Figure 3. Validated the regulators of 2C and ZGA genes

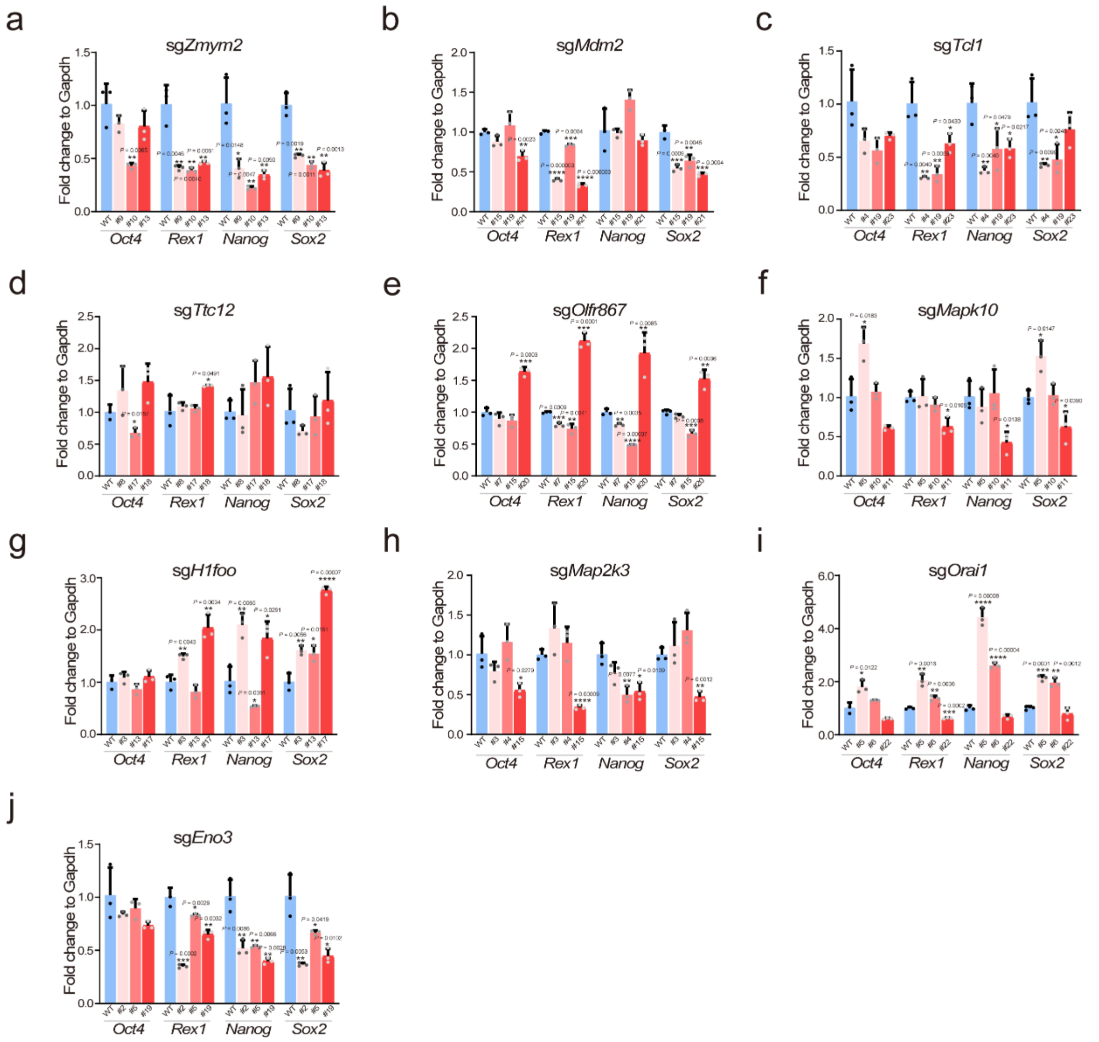

a-j. The relative expression of pluripotency genes in important candidate gene by RT-PCR. n = 3 biological replicates.

Data are analyzed by Student’s t -test. The data were mean ±SD. * P < 0.05, ** P < 0.01, *** P < 0.001 and ****P < 0.0001.

Supplementary Figure 4. The characterization of *Mdm2* KD mESCs

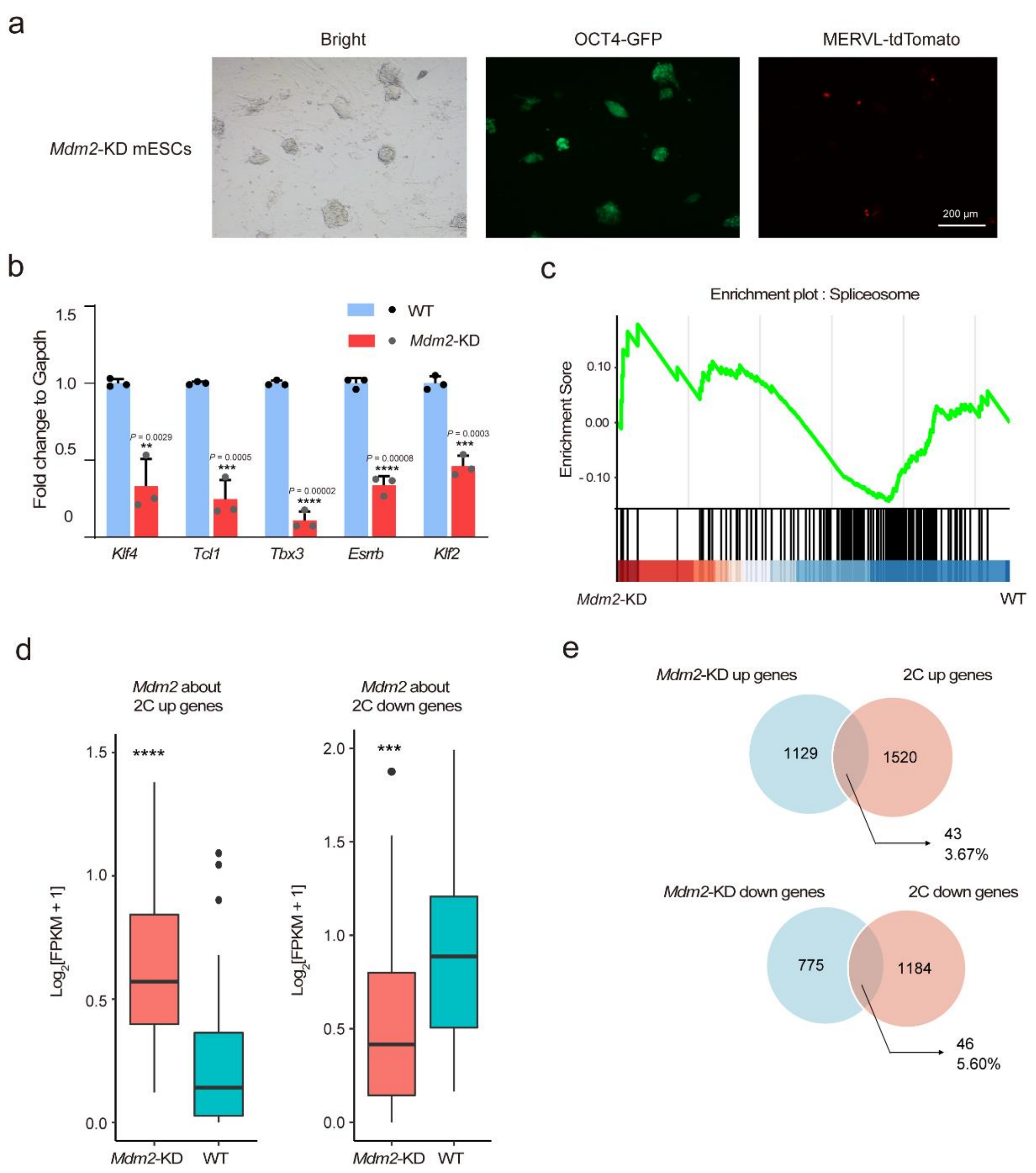

a. Microscope pictures(10x) of *Mdm2* KD mESCs.

b. The relative expression of important pluripotency genes in *Mdm2* KD mESCs by RT-PCR. n = 3 biological replicates. The data were mean ±SD.

c. GSEA analysis indicated down-regulation of spliceosome in *Mdm2* KD mESCs compared with WT mESCs.

d. The Veen diagram of overlapping genes of *Mdm2* regulatory genes and 2C regulatory

genes, respectively. *Mdm2* regulatory genes are defined by Log2FC >= 2 or Log2FC <= -2 with P < 0.05 in *Mdm2* KD mESCs compared to mESCs. 2C regulatory genes are defined by Log2FC >= 2 or Log2FC <= -2 with P < 0.05 in 2CLCs compared to mESCs.

e. The box plot showing different expression levels of *Mdm2* repressing about 2C up genes and *Mdm2* activating about 2C down genes.

Supplementary Figure 5. *Mdm2* influenced cell cycle to regulate 2C-Like state

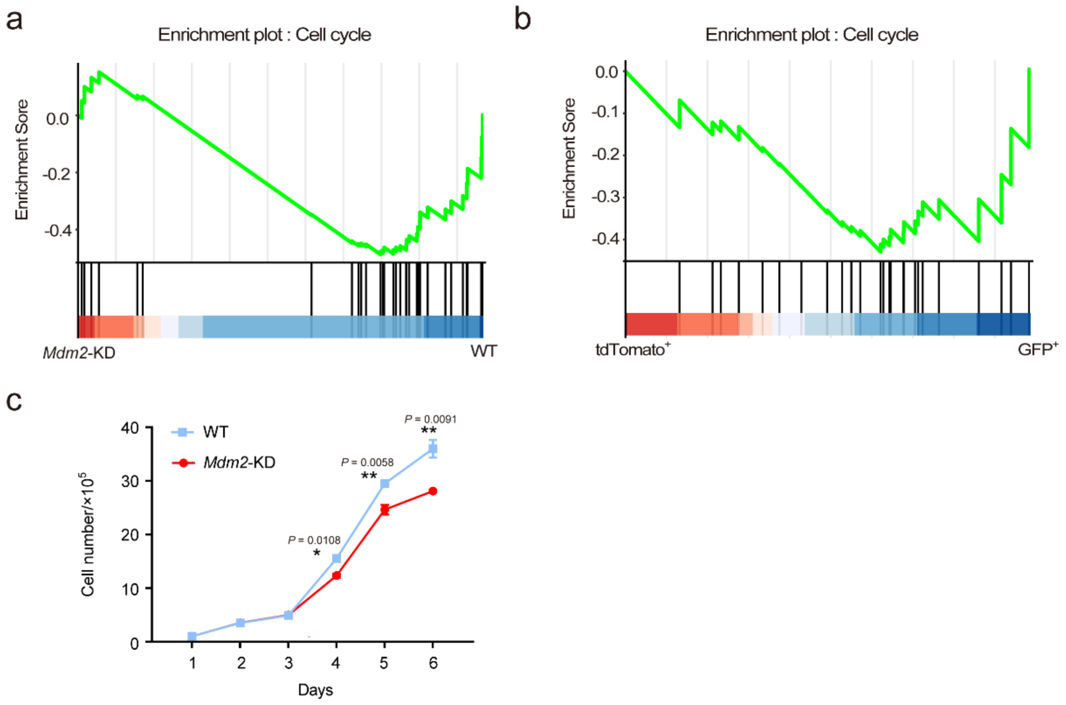

a. GSEA analysis of *Mdm2* KD and WT mESCs for the cell cycle gene set.

b. GSEA analysis of 2CLCs and mESCs for the cell cycle gene set.

c. Growth curves of *Mdm2* KD and WT mESCs. n = 3 biological replicates. The data were mean ±SD and performed by Student’s t -test. ** P < 0.01, *** P < 0.001 and ****P < 0.0001.

Supplementary Figure 6. H3K27me3 modifications influenced totipotency

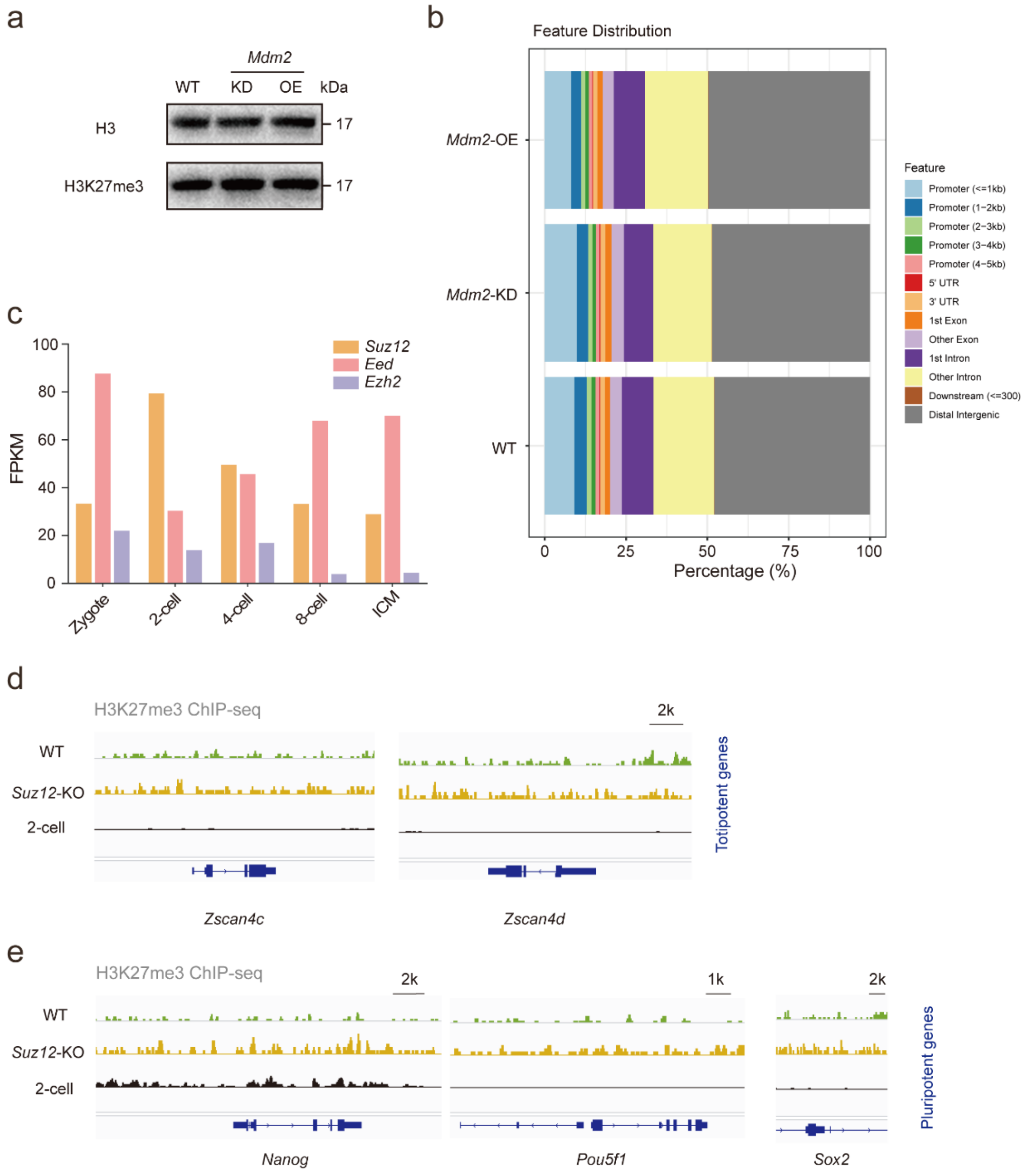

a. Western blot showed H3K27me3 expression level in the indicated mESCs with three similar results by western blot.

b. The distributions of differential H3K27me3 modifications in indicated mESCs.

c. *Suz12*, *Eed* and *Ezh2* expression level according to RNA-seq during in mouse pre-implantation embryos.

d. IGV view of H3K27me3 ChIP-seq results for 2C genes in *Suz12* KO, WT mESCs and

2C embryo.

e. IGV view of H3K27me3 ChIP-seq results for pluripotency genes in *Suz12* KO, WT mESCs and 2C embryo.

Supplementary Figure 7. The strategy for gating in the FACS plots.

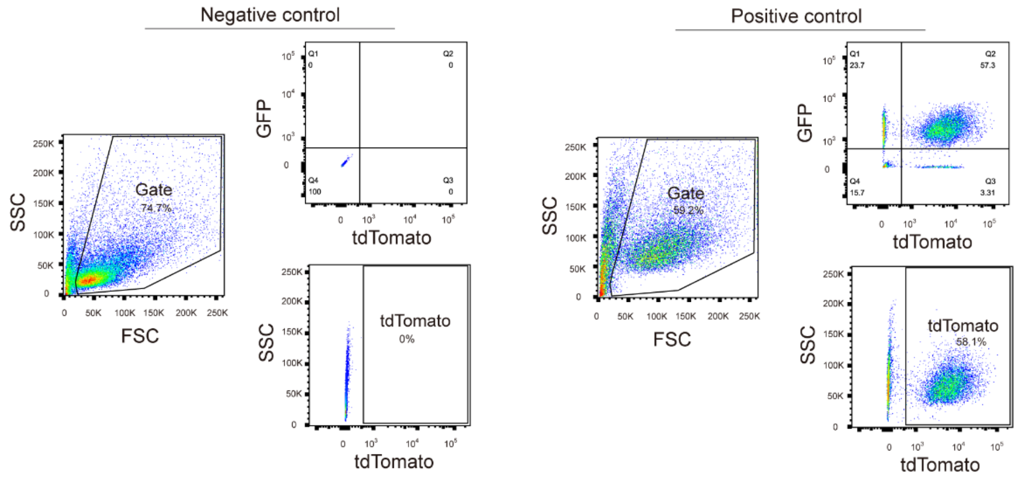

Supplementary Figure 8. The original and unedited blot images.

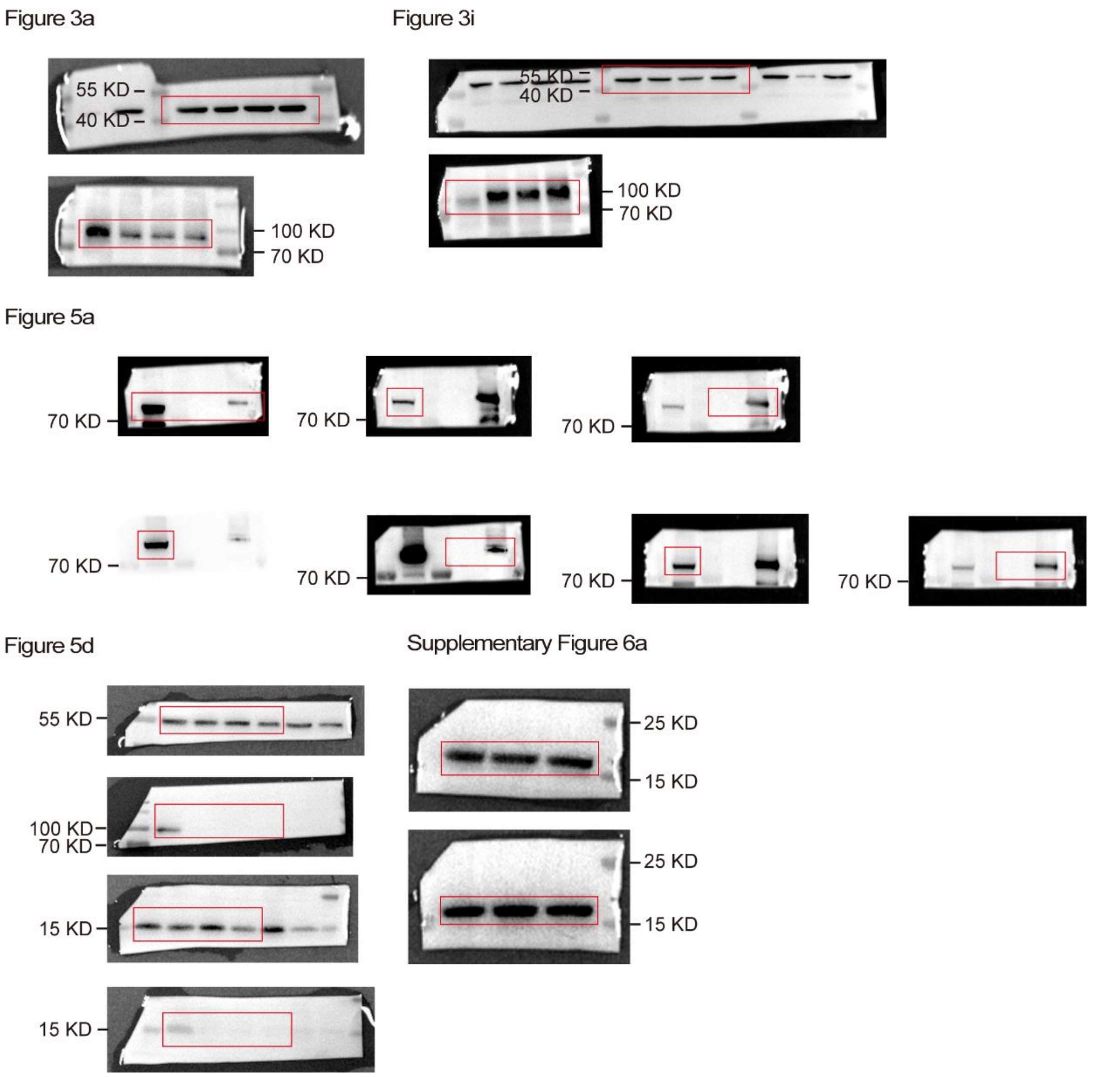

Supplementary Table 1. List of target genes and sgRNA sequence.

Gene	sgRNA sequence
Mdm2	AGTGTCGTTTTGCGCTCCAA
Mdm2	CGACACTTACACTATGAAAG
Mnt	CTTCGAATCTGAGCGTGCTG
Mnt	GAAGCGCAACATCCCCAACG
Uhrf1	TCCCATCCATAGTTCGAACC
Uhrf1	GGACAGAGAGTTCACGGTGT
Tcl1	TGGATGAGTTTCGTCGAAGC
Tcl1	CTGCACACCCCAACCGCCTG
Zmym2	GCAATTCATTATCAGACCAT
Zmym2	TTCGGTATCTGTTAAAGCAG
Dnmt1	CACCTAGTTCCGTGGCTACG
Dnmt1	CATCACGGCTCACTTCACGA
Map2k3	CACAGGTGGCTTGGACACGC
Map2k3	TCCCGGACCTTCATCACTAT
Mapk10	CTACTGCAGTGAACCAACCT
Mapk10	CACTGCAGTAGTATAAGAAA
Eno3	TGGAGACAAAGCACGATACC
Eno3	CATGCAAAAAATCTTCGCCC
Baz1a	TTCACCGAAAGCCTTTCGTG
Baz1a	AAACTTCCTCGTCCGGCCGC
H1foo	AGTTGCCGCAGAATCCAAGC
H1foo	CTGGAAACTGGCGTTCGTCG
Ddx49	TAGAACAGTGTCGACAGCTG
Ddx49	GATCGGGTTGTCGTCGTGGC
Thoc1	ACTGCCTTCAGTCAGTTACC
Thoc1	ACCTCTGAAAGCTTGGTCAA
Orai1	CCTCAACGAGCACTCGATGC
Orai1	TCCGGATTGCTGTGACTCGG
Olfr867	CATAGGAATCACACCCAGTA
Olfr867	CTATATAGAATGCCTTACAC
Zfp93	GACAACAGCCACATCCCTGA
Zfp93	GGAGAACTTCAGGAACCTCC
Slc26a4	CACCAGCCCAGTGCTAACTC
Slc26a4	CTCCGGAGATGATGTCACTG
Ppp2r2c	TGTATACATCGTACTCACCC
Ppp2r2c	GAGTGCGCGGCGTTCTGCTG
Suz12	AGAGTTCTGTGCAAAAATAT
Suz12	AGAAATCTATATATCTGTGT

Supplementary Table 2. List of RT-qPCR Primers.

List of RT-qPCR Primers	
**Primer Name**	**Sequence**
m*Dux*_qPCR_F	CCCAGCGACTCAAACTCCTTC
m*Dux*_qPCR _R	CCCTGCTGCCAGGATTTCTA
m*Zscan4*_qPCR_F	CCCTTCCTAGTGGTCGTGAATGTCTTT
m*Zscan4*_qPCR_R	CTGCTGTGAAGCCATTGTGGTGAC
m*Tcstv3*_qPCR F	ACCAGCTGAAACATCCATCC
m*Tcstv3*_qPCR R	CCATGGATCCCTGAAGGTAA
m*Gm4340*_qPCR F	CGAGGCACTGGGTCTAAGAG
m*Gm4340*_qPCR R	CCAATGAACAGGTCATGCTG
m*Oct4*_qPCR_F	TAGGTGAGCCGTCTTTCCAC
m*Oct4*_qPCR_R	GCTTAGCCAGGTTCGAGGAT
m*Sox2*_qPCR_F	ATTTAGAGCTAGACTCCGGGCGAT
m*Sox2*_qPCR_R	CTCCTTCCTTGTTTGTAACGGTCC
m*Nanog*_qPCR_F	TTGCTTACAAGGGTCTGCTACT
m*Nanog*_qPCR_R	ACTGGTAGAAGAATCAGGGCT
m*Rex1*_qPCR_F	TTGGGGCGAGCTCATTACTT
m*Rex1*_qPCR_R	TTGCCACACTCTGCACACAC
m*Actin*_qPCR_F	TAGGCACCAGGGTGTGATGG
m*Actin*_qPCR_R	CATGGCTGGGGTGTTGAAGG
m*Gadph*_qPCR_F	CTTCAACAGCAACTCCCACTC
m*Gadph*_qPCR_R	CCTGTTGCTGTAGCCGTATTC
