# Supplementary material for: A CRISPR/Cas9 screen in embryonic stem cells reveals that Mdm2 regulates totipotency exit
Source: Commun Biol. 2024 Jul 3;7:809. doi: 10.1038/s42003-024-06507-9 (PMC11222520; doi:10.1038/s42003-024-06507-9)
Supplement: Supplementary file 2 — Description of Additional Supplementary Files [file 42003_2024_6507_MOESM2_ESM.pdf]

Description of Additional Supplementary Files

File name: Supplementary Data 1

Description: This Excel file contains the source data used to generate the graphs in the paper.
